# Supplementary material for: Transcriptomic divergence of network hubs in the prenatal human brain
Source: Commun Biol. 2025 Nov 18;8:1597. doi: 10.1038/s42003-025-08962-4 (PMC12627746; doi:10.1038/s42003-025-08962-4)
Supplement: Supplementary file 2 — Supplemental Material [file 42003_2025_8962_MOESM2_ESM.pdf]

SUPPLEMENTAL MATERIAL

Transcriptomic divergence of network hubs in the prenatal human brain

S. Oldham[1] & G. Ball[1,2]
1. Developmental Imaging, Murdoch Children's Research Institute
2. Turner Institute for Brain and Mental Health, Monash University
3. Department of Paediatrics, University of Melbourne

gareth.ball@mcri.edu.au

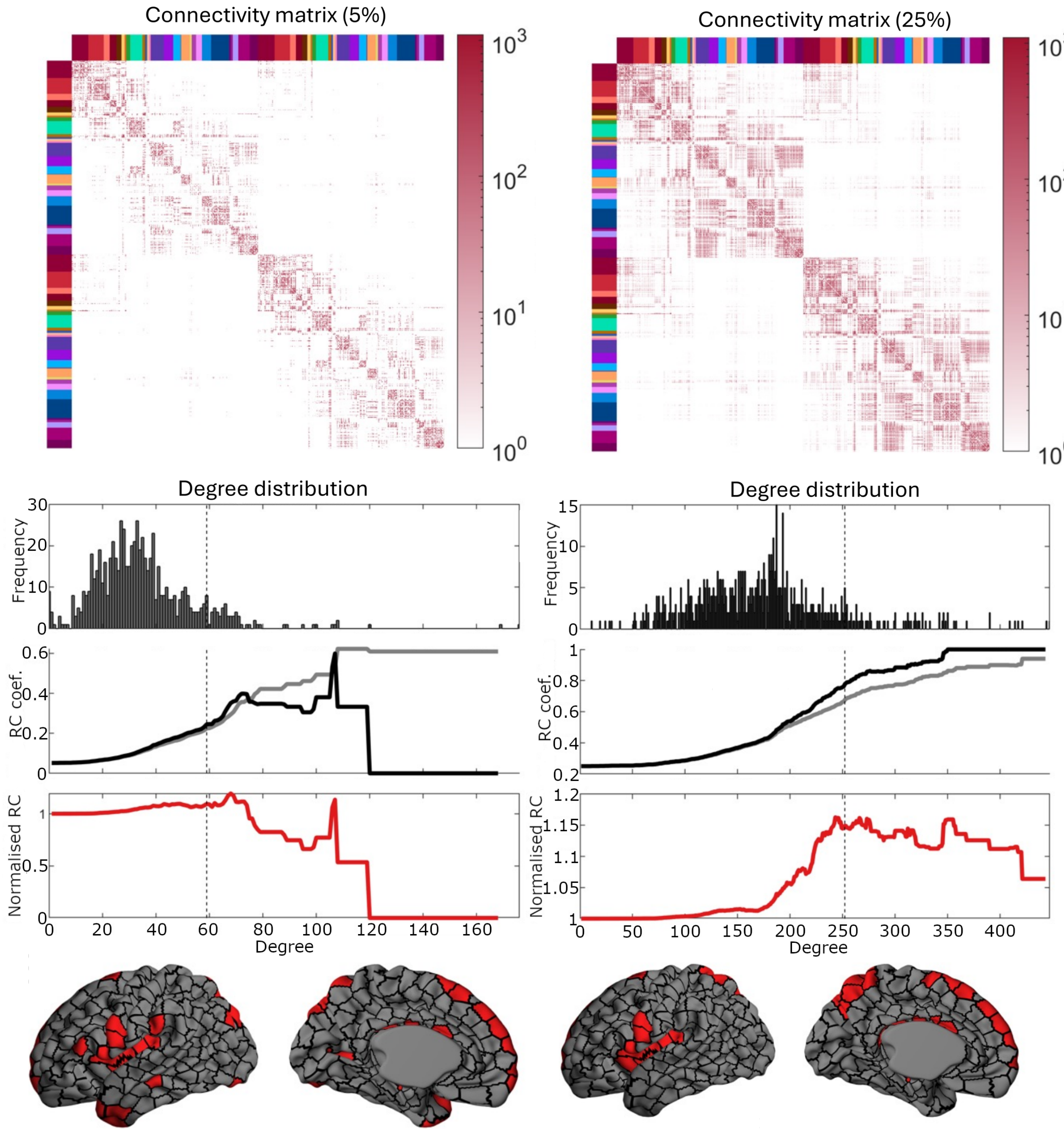

Figure S1. Alternative network thresholds. Group consensus connectivity matrices thresholded at two alternative levels (retaining the strongest 5%, 25% of all edges) are shown (top row) for the μBrain$_{90}$ parcellation. Corresponding degree distributions and rich club coefficient (RC) curves (middle row) are shown for each thresholded consensus network. The rich-club coefficient is calculated over degree thresholds in the empirical data (black line) and compared to the rich-club coefficient calculated in degree sequence preserving null networks (grey line). Normalised rich-club coefficient (red line) values > 1 indicate greater rich-club organisation than expected by chance. The dashed vertical line indicates the 90th percentile for node degree to define network hubs. The location of hub nodes across hemispheres (bottom row; coloured regions) in each consensus network are shown overlaid on the corresponding cortical parcellation.

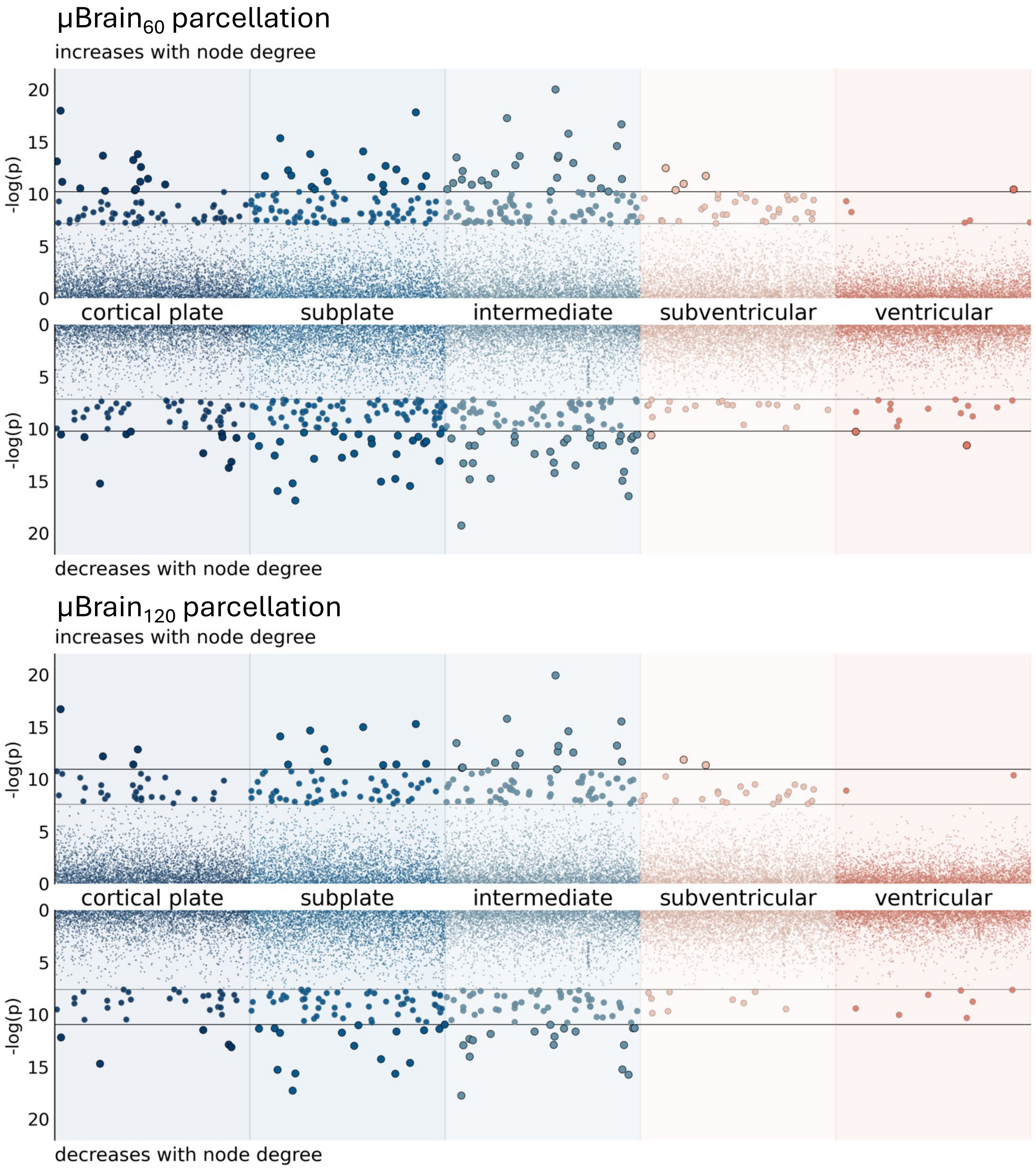

Figure S2: Significant associations between node degree and gene expression at different network resolutions. Associations are shown for two alternative parcellations (μBrain$_{60}$ and μBrain$_{120}$). Each marker represents a significant gene association. Colours indicate different tissue zones, dashed and dotted lines illustrate p_{FDR}<0.05 and p_{FDR}<001, respectively.
