# Supplementary material for: Transcriptomic divergence of network hubs in the prenatal human brain
Source: Commun Biol. 2025 Nov 18;8:1597. doi: 10.1038/s42003-025-08962-4 (PMC12627746; doi:10.1038/s42003-025-08962-4)
Supplement: Supplementary file 3 — Description of Additional Supplementary Files [file 42003_2025_8962_MOESM3_ESM.docx]

**Description of Additional Supplementary Files**

**File name:** Supplementary Data 1

**Description:** Average node degree and proportion of rich club nodes in each cortical region

**File name:** Supplementary Data 2

**Description:** Significant associations between gene expression and node degree in each developmental tissue zone

**File name:** Supplementary Data 3

**Description:** Cell lineage enrichment for hub+ and hub- genes expressed in each tissue zone

**File name:** Supplementary Data 4

**Description:** Gene set enrichment for hub+ and hub- genes

**File name:** Supplementary Data 5

**Description:** MAGMA analysis of hub+ and hub- genes

**File name:** Supplementary Data 6

**Description:** Source data underlying main figures
