# Supplementary material for: Transcriptomic divergence of network hubs in the prenatal human brain
Source: Commun Biol. 2025 Nov 18;8:1597. doi: 10.1038/s42003-025-08962-4 (PMC12627746; doi:10.1038/s42003-025-08962-4)
Supplement: Supplementary file 6 — Reporting summary [file 42003_2025_8962_MOESM6_ESM.pdf]

nature portfolio

Corresponding author(s): Ball
Last updated by author(s): Jun 30, 2025

Reporting Summary

Nature Portfolio wishes to improve the reproducibility of the work that we publish. This form provides structure for consistency and transparency in reporting. For further information on Nature Portfolio policies, see our Editorial Policies and the Editorial Policy Checklist.

Statistics

For all statistical analyses, confirm that the following items are present in the figure legend, table legend, main text, or Methods section.

n/a	Confirmed	
☐	☒	The exact sample size (*n*) for each experimental group/condition, given as a discrete number and unit of measurement
☐	☒	A statement on whether measurements were taken from distinct samples or whether the same sample was measured repeatedly
☐	☒	The statistical test(s) used AND whether they are one- or two-sided<br>*Only common tests should be described solely by name; describe more complex techniques in the Methods section.*
☐	☒	A description of all covariates tested
☐	☒	A description of any assumptions or corrections, such as tests of normality and adjustment for multiple comparisons
☐	☒	A full description of the statistical parameters including central tendency (e.g. means) or other basic estimates (e.g. regression coefficient) AND variation (e.g. standard deviation) or associated estimates of uncertainty (e.g. confidence intervals)
☐	☒	For null hypothesis testing, the test statistic (e.g. *F*, *t*, *r*) with confidence intervals, effect sizes, degrees of freedom and *P* value noted<br>*Give P values as exact values whenever suitable.*
☒	☐	For Bayesian analysis, information on the choice of priors and Markov chain Monte Carlo settings
☒	☐	For hierarchical and complex designs, identification of the appropriate level for tests and full reporting of outcomes
☐	☒	Estimates of effect sizes (e.g. Cohen's *d*, Pearson's *r*), indicating how they were calculated

Our web collection on statistics for biologists contains articles on many of the points above.

Software and code

Policy information about availability of computer code

Data collection	n/a
Data analysis	Structural images were processed using the dHCP's minimal preprocessing pipeline, including bias correction, brain extraction, tissue segmentation and cortical surface reconstruction. Whole-brain tractography was performed using MRtrix3 (v3.0.2).To generate brain networks from the whole brain tractograms, we first applied Connectome Spatial Smoothing (CSS). Network analysis was performed in Matlab. Other data analysis was performed in Python using numpy, scipy, sklearn, statsmodels & pandas libraries. GO enrichment was performed using gene2func implemented in FUMA.90 SNP enrichment was performed using MAGMA. Supporting code is available at https://github.com/garedaba/prenatal-hubs

For manuscripts utilizing custom algorithms or software that are central to the research but not yet described in published literature, software must be made available to editors and reviewers. We strongly encourage code deposition in a community repository (e.g. GitHub). See the Nature Portfolio guidelines for submitting code & software for further information.

Data

Policy information about availability of data

All manuscripts must include a data availability statement. This statement should provide the following information, where applicable:

- Accession codes, unique identifiers, or web links for publicly available datasets
- A description of any restrictions on data availability
- For clinical datasets or third party data, please ensure that the statement adheres to our policy

Neuroimaging data for the Developing Human Connectome Project are available via the NIMH Data Archive (collection ID: 3955). The µBrain atlas and associated data is available at: https://zenodo.org/records/10622337. Harmonised and log-normalised single-nucleus RNA sequencing profiles for 709,372 cells were downloaded from the UCSC Cell Browser (cells.ucsc.edu/?ds=pre-postnatal-cortex)

Research involving human participants, their data, or biological material

Policy information about studies with human participants or human data. See also policy information about sex, gender (identity/presentation), and sexual orientation and race, ethnicity and racism.

Reporting on sex and gender	Group average network were used in this analysis. Sex differences in network connectivity were not considered. Transcriptomic data were not analysed by sex due to small sample size
Reporting on race, ethnicity, or other socially relevant groupings	n/a
Population characteristics	Participant data was acquired from the third release of the Developing Human Connectome Project (dHCP). Ethics approval was granted by the United Kingdom Health Research Ethics Authority, reference no. 14/LO/1169. The full cohort comprised 783 neonates (360 female; median birth age [range] = 39+2 weeks [23-43+4]) across 889 scans (median scan age [range] = 40 +6 [26+5-45+1] weeks; 107 neonates were scanned multiple times). For this study, only neonatal scans acquired from term-born infants with a radiological score of 1 or 2 (indicating no/minimal radiological abnormalities or pathologies) that also met additional quality control for in-scanner motion criteria (see below) where included, resulting in a final cohort of 208 neonates (101 females; median gestational age at birth [range] = 40+1 weeks [37- 42+2]; median post-menstrual age at scan [range] = 41 [37+3 – 44+5] weeks).
Recruitment	Participant data was acquired from the third release of the Developing Human Connectome Project (dHCP).
Ethics oversight	Ethics approval for dHCP data collection and dissemination was granted by the United Kingdom Health Research Ethics Authority, reference no. 14/LO/1169.

Note that full information on the approval of the study protocol must also be provided in the manuscript.

Field-specific reporting

Please select the one below that is the best fit for your research. If you are not sure, read the appropriate sections before making your selection.

☒ Life sciences ☐ Behavioural & social sciences ☐ Ecological, evolutionary & environmental sciences

For a reference copy of the document with all sections, see nature.com/documents/nr-reporting-summary-flat.pdf

Life sciences study design

All studies must disclose on these points even when the disclosure is negative.

Sample size	Participant data was acquired from the third release of the Developing Human Connectome Project (dHCP). The full cohort comprised 783 neonates (360 female; median birth age [range] = 39+2 weeks [23-43+4]) across 889 scans (median scan age [range] = 40+6 [26+5-45+1] weeks; 107 neonates were scanned multiple times). After exclusions, this resulted in a final cohort of 208 neonates (101 females; median gestational age at birth [range] = 40+1 weeks [37- 42+2]; median post-menstrual age at scan [range] = 41 [37+3 – 44+5] weeks).
Data exclusions	For this study, only neonatal scans acquired from term-born infants with a radiological score of 1 or 2 (indicating no/minimal radiological abnormalities or pathologies) that also met additional quality control for in-scanner motion criteria where included,
Replication	A number of sensitivity analyses were performed to test whether network fndings replicaated over different parameter settings. Enrichment of hub genes during prenatal periods were replicated in an independent RNA-seq dataset.
Randomization	n/a
Blinding	n/a

Reporting for specific materials, systems and methods

We require information from authors about some types of materials, experimental systems and methods used in many studies. Here, indicate whether each material, system or method listed is relevant to your study. If you are not sure if a list item applies to your research, read the appropriate section before selecting a response.

Materials & experimental systems

n/a	Involved in the study
☒	☐ Antibodies
☒	☐ Eukaryotic cell lines
☒	☐ Palaeontology and archaeology
☒	☐ Animals and other organisms
☒	☐ Clinical data
☒	☐ Dual use research of concern
☒	☐ Plants

Methods

n/a	Involved in the study
☒	☐ ChIP-seq
☒	☐ Flow cytometry
☐	☒ MRI-based neuroimaging

Plants

Seed stocks

Report on the source of all seed stocks or other plant material used. If applicable, state the seed stock centre and catalogue number. If plant specimens were collected from the field, describe the collection location, date and sampling procedures.

Novel plant genotypes

Describe the methods by which all novel plant genotypes were produced. This includes those generated by transgenic approaches, gene editing, chemical/radiation-based mutagenesis and hybridization. For transgenic lines, describe the transformation method, the number of independent lines analyzed and the generation upon which experiments were performed. For gene-edited lines, describe the editor used, the endogenous sequence targeted for editing, the targeting guide RNA sequence (if applicable) and how the editor was applied.

Authentication

Describe any authentication procedures for each seed stock used or novel genotype generated. Describe any experiments used to assess the effect of a mutation and, where applicable, how potential secondary effects (e.g. second site T-DNA insertions, mosiacism, off-target gene editing) were examined.

Magnetic resonance imaging

Experimental design

Design type: n/a

Design specifications: n/a

Behavioral performance measures: n/a

Acquisition

Imaging type(s): Diffusion

Field strength: 3

Sequence & imaging parameters: Images were acquired on a Phillips Achieva 3T scanner at St Thomas Hospital, London, United Kingdom using a dedicated neonatal imaging system. T2-weighted Fast Spin Echo (FSE) multislice images were acquired in sagittal and axial orientations with overlapping slices (TR = 12000 ms; TE = 156 ms; resolution = 0.8 × 0.8 × 1.6 mm, 0.8 mm overlap). Sagittal and axial image stacks were motion corrected and reconstructed into a single 3D volume. Diffusion MRI was acquired with a spherically optimised set of directions over 4 b-shells (20 volumes × b = 0 s/mm2; 64 directions × b = 400; 88 × b = 1,000; 128 × b = 2,600)155,156 with a multiband factor acceleration of 4, TR = 3,800 ms; TE = 90 ms; SENSE: 1.2 and acquired resolution of 1.5 mm × 1.5 mm with 3-mm slices (1.5-mm overlap) reconstructed using an extended SENSE technique into 1.5 × 1.5 × 1.5 mm volumes.

Area of acquisition: whole-brain

Diffusion MRI ☒ Used ☐ Not used

Parameters: Diffusion MRI was acquired with a spherically optimised set of directions over 4 b-shells (20 volumes × b = 0 s/mm2; 64 directions × b = 400; 88 × b = 1,000; 128 × b = 2,600)155,156 with a multiband factor acceleration of 4, TR = 3,800 ms; TE = 90 ms; SENSE: 1.2 and acquired resolution of 1.5 mm × 1.5 mm with 3-mm slices (1.5-mm overlap) reconstructed using an extended SENSE technique into 1.5 × 1.5 × 1.5 mm volumes.

Preprocessing

Field	Response
Preprocessing software	Structural images were processed using the dHCP's minimal preprocessing pipeline, including bias correction, brain extraction, tissue segmentation and cortical surface reconstruction. Diffusion data were processed using a neonatal-specific pipeline. This included correction of susceptibility and eddy-current-induced distortions, motion artefacts and signal dropout, automated extraction of quality control (QC) metrics and nonlinear alignment to the 40-week dHCP neonatal template via each subject's corresponding anatomical data.
Normalization	Nonlinear alignment to the 40-week dHCP neonatal template via each subject's corresponding anatomical data.
Normalization template	40-week dHCP neonatal template
Noise and artifact removal	To ensure only high-quality scans without major motion-related artefacts were used, we examined the quality control summaries of the dHCP diffusion processing pipeline. Scans more than two standard deviations away from the mean on any of the volume- to-volume motion, within-volume motion, susceptibility-induced distortions, and eddy current-induced distortions metrics were excluded from further analysis.
Volume censoring	n/a

Statistical modeling & inference

Field	Response
Model type and settings	For each gene, we used a General Linear Model (GLM) to test the hypothesis that mid-gestation gene expression was associated with node degree (total number of connections) in each cortical region. GLMs were performed for each gene in each of 5 tissue zones (total number of tests = 7457 per zone) including the age of each specimen (15/16 or 21PCW) as a covariate. Robust linear models were fit to expression data using iteratively reweighted least squares to account for potential outliers
Effect(s) tested	Linear association between node degree and gene expression in each network node.

Specify type of analysis: ☐ Whole brain ☒ ROI-based ☐ Both

Anatomical location(s): *Describe how anatomical locations were determined (e.g. specify whether automated labeling algorithms or probabilistic atlases were used).*

Field	Response
Statistic type for inference (See Eklund et al. 2016)	n/a
Correction	Significant associations between expression and node degree were identified after multiple comparison correction for False Discovery Rate within each tissue zone (pFDR<0.05)

Models & analysis

n/a	Involved in the study
☒	☐ Functional and/or effective connectivity
☐	☒ Graph analysis
☒	☐ Multivariate modeling or predictive analysis

Graph analysis

Hubs were defined as previously described in adult data. We initially constructed a group consensus network by selecting connections that were (i) present in over 30% of individual networks, and (ii) in the top 15% of connections by strength. To identify hubAs nodes with a higher degree are more likely to be connected to each other by chance, we generated 1000 random networks computed the rich-club coefficient ϕ_rand (k) for each of these networks. The random networks were created by rewiring each edge of the group consensus network 50 times while retaining the degree sequence of the original network. The normalised rich-club coefficients were calculated as the ratio between the group consensus rich-club coefficient and the mean rich-club coefficient across the random networks for a given degree threshold, ks within the consensus network, the rich-club coefficient was calculated across k degree thresholds .
